# Cost Effectiveness of Hand Postaxial Polydactyly Type B Excision in the Office Versus Operating Room

**DOI:** 10.1016/j.jhsg.2025.100865

**Published:** 2025-10-30

**Authors:** Manisha Banala, Sarah L. Struble, Holly Cordray, Benjamin Chang, Apurva S. Shah, Eliza Buttrick, John R. Vaile, Shaun D. Mendenhall

**Affiliations:** ∗ Perelman School of Medicine at the University of Pennsylvania, Philadelphia, PA; † Department of Plastic Surgery, University of California Irvine, Irvine, CA; ‡ Division of Plastic, Reconstructive, and Oral Surgery, Children’s Hospital of Philadelphia, Philadelphia, PA; § Division of Orthopaedic Surgery, Children’s Hospital of Philadelphia, Philadelphia, PA; ‖ Division of Plastic Surgery, Department of Surgery, Lehigh Valley Health Network, Allentown, PA; ¶ Division of Plastic & Reconstructive Surgery, Department of Surgery, Spencer Fox Eccles School of Medicine at the University of Utah and Intermountain Primary Children's Hospital, Salt Lake City, UT

**Keywords:** Congenital hand, Cost analysis, Pediatric hand surgery, Polydactyly excision

## Abstract

**Purpose:**

Postaxial polydactyly type B of the hand can be excised in the office with local anesthesia or in the operating room under general anesthesia. Existing studies have not described the cost difference between these treatments. We compared charges, reimbursements, and outcomes of office and operating room excision.

**Methods:**

All cases of postaxial polydactyly type B excision at a children’s hospital from fiscal year 2018–2023 were included. Independent *t* tests and Fisher exact tests were performed to compare the charges and outcomes of office and operating room excisions. Random-effects models were used to compare differences in reimbursements since these data were obtained as summary statistics (mean [SD]) by fiscal year.

**Results:**

Of 620 total patients, 30 (4.8%) underwent operating room excisions (22 bilateral and 8 unilateral), and 590 (95%) had office excisions (346 bilateral and 244 unilateral). Average total charges were 78% and 84% less for bilateral and unilateral excisions, respectively, in the office than in the operating room. Insurance companies paid $7800 more for bilateral operating room excisions and $7600 more for unilateral operating room excisions. Surgeons received $2300 more for bilateral operating room excisions and $1300 more for unilateral operating room excisions. The treatment strategy did not impact outcomes.

**Conclusions:**

Given the cost efficacy and safety of office excision, operating room excision should be limited to patient-specific factors requiring general anesthesia.

**Type of study/level of evidence:**

Prognosis IIb.

Postaxial polydactyly type B (PPB) is characterized by a nonfunctional supernumerary digit attached to the small finger by a thin skin/neurovascular pedicle, and it is often treated by either ligation or surgical excision.^1^^,^^2^ Initially, ligation was the preferred treatment strategy, as it could be performed by a primary care physician without local or general anesthesia.^2^ It involves tying off the base of the skin bridge with a suture or metal clip to induce ischemic necrosis and autoamputation.^2^ However, this can lead to parental anxiety related to the necrotic autoamputation process, an unsightly residual bump, or a symptomatic neuroma.^2^, ^3^, ^4^, ^5^ After studies revealed the ligation’s complication rate, surgical excision became the treatment of choice.^6^, ^7^, ^8^, ^9^, ^10^ Surgical excision involves an elliptical incision at the base of the supernumerary digit, followed by traction neurectomy of the accessory digital nerve.^11^ In the office, it is often performed with local anesthesia for newborns. In the operating room, it is often delayed until the patient is at least 6 months old to diminish the risks associated with general anesthesia use in infants.^12^ Previous studies have shown operating room excisions have similar outcomes to office excisions but higher billing charges, lengthier postoperative discharge processes, and longer hospital stays.^7^^,^^13^^,^^14^ Despite these findings, surgeon preference and training appear to be the primary drivers of treatment location. In a previous study, 29% of the 59 hand surgeons surveyed preferred operating room excision.^11^ Further comparisons of both costs and outcomes associated with each strategy will therefore help guide treatment guidelines. Moreover, to our knowledge, no existing studies have analyzed the difference in reimbursements. To thoroughly compare both treatment strategies, this study analyzed charges, reimbursements, and outcomes of PPB operating room and office surgical excisions. We hypothesized that operating room excisions have similar outcomes, but higher total charges and reimbursements than office excisions.

## Materials and Methods

With institutional review board approval from the Children’s Hospital of Philadelphia (21-019312), this study reviewed all procedures booked with current procedural terminology (CPT) codes 26587 (“reconstruction of polydactylous digit, soft tissues, and bone”), 11420 (“excision of a benign lesion”), 11200 (“removal of skin tags, up to 15 lesions”), and 64774 (“excision of neuroma; cutaneous nerve, surgically identifiable”) at a tertiary, 627-bed academic pediatric hospital from fiscal year (FY) 2018–2023. Of this cohort, this study identified and included all patients who underwent unilateral or bilateral hand PPB surgical excision either in the office or in the operating room. Patients who underwent operating room excision to simultaneously address another surgical need were excluded from the analysis.

Demographics, treatment strategy, and complications data were extracted from patient records, whereas charge, reimbursement, and CPT code data were collected from the hospital’s finance department. Office excisions only incurred surgeon charges (no facility or anesthesia charges applied), but operating room excisions also included associated hospital charges (facilities, anesthesia, etc). Reimbursements were specific to the payor mix and insurance coverage of the patients analyzed and are subject to change for other populations. Charges and reimbursements before FY2023 were adjusted for inflation using the Consumer Price Index and reported in FY2023 USD.

Descriptive statistics were conducted to describe the demographics of the cohort of patients included. Independent sample *t* tests and Fisher exact tests were used to compare charges and outcomes of office and operating room excisions. *P* values less than .05 were considered statistically significant. Reimbursement data were obtained as summary statistics (mean [SD]) by FY to preserve insurance company privacy. Therefore, primary data were combined and compared using the “meta” package for R Statistical Software (version 4.1.3),^15^^,^^16^ which provides a valid method for handling summary statistics with different sample sizes. Analyses compared mean differences in costs for operating room versus office procedures, stratifying by payor and excision type (unilateral or bilateral). Data were combined in random-effects models with the Hartung-Knapp modification^17^ and inverse-variance weighting to determine mean differences with 95% CIs and Hedges *g* effect sizes (the standardized mean difference). A Hedges *g* of 0.2 represents a small effect, 0.5 is a medium effect, and 0.8 is a large effect.

## Results

Of 620 total patients, 30 (4.8%) underwent operating room excisions (22 bilateral and 8 unilateral), and 590 (95%) underwent office excisions (346 bilateral and 244 unilateral; Table 1). The cohort had a 1.3:1.0 man-to-woman ratio and primarily identified as Black (72%; Table 1). Median age at excision was 12.2 (6.1–171.0) months in the operating room and 1.1 (0.1–15.0) months in the office (Table 1).

**Table 1:** Demographic and Clinical Characteristics of Type B Polydactyly Patients (n = 620)

Excision Type	n (%)
Operating room, bilateral	22 (3.5)
Operating room, unilateral	8 (1.3)
Office, bilateral	346 (55.8)
Office, unilateral	244 (39.4)
Sex (n [%])	
Male	348 (56.1)
Female	272 (43.9)
Race (n [%])	
Black	444 (71.6)
Other	100 (16.1)
White	76 (12.3)
Age at excision, mo (median [range])	
Operating room	12.2 (6.1–171.0)
Clinic	1.1 (0.1–15.0)

Average total insurance payments ranged from 15% of average total charges in FY2018 to 28% in FY2020 and FY2022 for unilateral PPB office excision. For bilateral PPB office excision, they ranged from 10% in FY2018 to 22% in FY2019 and FY2022. For unilateral PPB operating room excision, average total insurance payments ranged from 16% of average total charges in FY2018 to 39% in FY2019. For bilateral PPB operating room excision, they ranged from 18% in FY2023 to 43% in FY2020.

Compared with operating room procedures, average total charges for office procedures were 78% and 84% less for bilateral and unilateral excisions, respectively (*P* < .001; Table 2). Surgeon fees for office excisions were 40% less than those for operating room excisions (bilateral *P* < .001, unilateral *P* = .002; Table 2).

**Table 2:** Average Billing Charges for Type B Polydactyly Excision

Charge and Excision Type	Operating Room Average ($ [SD])	Office Average ($ [SD])	Difference (%)	*P*
Hospital, bilateral	24.1 k (±5.2 k)	-	100	-
Surgeon, bilateral	13.7 k (±2.6 k)	8.4 k (±4.9 k)	39	<.001
Total	37.9 k (±6.4 k)	8.4 k (±4.9 k)	78	<.001
Hospital, unilateral	18.4 k (±2.7 k)	-	100	-
Surgeon, unilateral	6.8 k (±1.8 k)	4.0 k (±2.4 k)	40	.002
Total	25.2 k (±3.5 k)	4.0 k (±2.4 k)	84	<.001

Insurance companies paid more for operating room excisions than office excisions, although cost differences for patients were not statistically significant (Table 3). On average, insurance companies paid $7.8 k more when bilateral excisions were performed in the operating room (CI, $5.5 k to $10.2 k; g = 3.28; *P* < .001) and $7.6 k more when unilateral excisions were performed in the operating room (CI, $4.5 k to $10.7 k; g = 5.29; *P* < .001). Surgeon reimbursements were also higher for operating room excisions (Table 4). Compared with office excisions, surgeons received an average of $2.3 k (CI, $0.7 k to $3.9 k; g = 1.06; *P* = .005) more for bilateral operating room excisions and $1.3 k (CI, $0.8 k to $1.8 k; g = 0.71; *P* < .001) more for unilateral operating room excisions.

**Table 3:** Insurance and Patient Payments for Operating Room Versus Office Excisions

Payor	Excision Type	Operating Room, n	Office, n	Mean Difference∗	95% CI	Hedges *g*	*P*
Insurance	Bilateral	22	346	$7.8 k	($5.5 k to $10.2 k)	3.28	<.001
	Unilateral	8	243	$7.6 k	($4.5 k to $10.7 k)	5.29	<.001
Patient	Bilateral	22	346	$0.4 k	(−$0.2 k to $1.1 k)	-	.19
	Unilateral	8	243	$0.2 k	(−$0.2 k to $0.5 k)	-	.33

∗ Mean amount by which operating room payments exceeded office payments.

**Table 4:** Surgeon Reimbursements for Operating Room Versus Office Excisions

Excision Type	Operating Room, n	Office, n	Mean Difference∗	95% CI	Hedges *g*	*P*
Bilateral	22	346	$2.3 k	($0.7 k to $3.9 k)	1.06	.005
Unilateral	8	243	$1.3 k	($0.8 k to $1.8 k)	0.71	<.001

∗ Mean amount by which operating room reimbursements exceeded office reimbursements.

One operating room patient (3.3%) and eight office patients (1.4%) had complications (Table 5). The operating room patient had a surgical site infection. Among the office excision patients with complications, three had surgical site infections, three experienced persistent pain/neuroma, and two presented with hypertrophic scarring. Notably, the treatment strategy did not significantly impact outcomes (*P* = .4; Table 5).

**Table 5:** Incidence of Postoperative Complications for Type B Polydactyly Excision (Operating Room, n = 30; Office, n = 590)

Complication	Operating Room (n [%])	Office (n [%])	*P*
Infection	1 (3.3)	3 (0.5)	.2
Persistent pain/neuroma	0 (0)	3 (0.5)	>.99
Hypertrophic scarring	0 (0)	2 (0.3)	>.99
Total	1 (3.3)	8 (1.4)	.4

*P* values are from Fisher exact tests.

Current procedural terminology code usage for PPB excision varied. In the operating room, 90% of procedures were booked with code 26587 or “reconstruction of polydactylous digit, soft tissues, and bone,” 6.7% with 11420 or “excision of a benign lesion,” and 3.3% with 11200 or “removal of skin tags, up to 15 lesions.” Of the office procedures, 63% were booked with code 26587, 37% with 11420, and 0.2% with 11200.

## Discussion

This study analyzed charges, reimbursements, outcomes, and CPT billing trends of operating room and office PPB excisions to thoroughly compare the cost difference between these treatment strategies. It presents evidence of cost effectiveness of office surgical excision in treating PPB. Our findings provide valuable additions to previous efforts to illuminate the cost difference and outcomes between these two excision methods and contribute to ongoing efforts to develop treatment guidelines for PPB.^2^, ^3^, ^4^, ^5^, ^6^, ^7^, ^8^, ^9^, ^10^ Additionally, our analysis presents an overview of CPT billing trends for these procedures, adding more context to previously published survey results about surgeons’ CPT code preferences regarding PPB excision.^11^

In our cohort, there was no considerable difference in outcomes between operating room and office surgical excisions. In 1985, Simmons recommended that PPB excision be performed under general anesthesia when a child is older than 6 months.^18^ However, since then, multiple studies have highlighted the success of office excisions using local anesthesia, even in older children.^8^^,^^19^, ^20^, ^21^ Some studies have also directly compared office and operating room excisions and reported that they have similar complication rates.^7^^,^^22^ Therefore, for clinically standard presentations of PPB, our study confirms that surgeons and patients can expect similar outcomes from office and operating room excision.

Consistent with previous studies, operating room excisions had higher billing charges than office excisions.^7^^,^^13^^,^^14^ As hospitals must account for additional expenses associated with operating room procedures, like general anesthesia and operating room facilities, this result was expected. Our analysis also confirmed that, likely because of increased usage of CPT code 26587 in the operating room, insurance companies paid more, while surgeons earned higher reimbursements, for operating room excisions. To our knowledge, no previous studies have compared reimbursements of operating room and office PPB excision. Despite our findings, patients could still present with factors warranting operating room excision, like having a relatively large soft tissue bridge connecting the polydactylous digit to the small finger or being unable to remain still for an office procedure. Other potential reasons surgeons may opt for operating room excision include parental anxiety, insufficient office infrastructure, or other conditions requiring general anesthesia. These factors should be evaluated on a case-by-case basis, allowing surgeons to exercise their clinical judgment in determining when a case is not suitable for an office procedure.

Although CPT code 26587 (“reconstruction of polydactylous digit, soft tissues, and bone”) was used for 90% of operating room procedures, there was more variability in office procedure billing. Of the office procedures analyzed, 63% were billed with code 26587, 37% with 11420 (“excision of a benign lesion”), and 0.2% with 11200 (“removal of skin tags, up to 15 lesions”). Per CPT guidelines, the 26587 code applies to procedures including excision or reconstruction of bone as well as soft tissue.^23^ As PPB excision does not involve bone reconstruction, surgeons’ interpretations of the code have differed. In 2016, Carpenter et al stated that CPT code 26587 was inappropriate for PPB excision.^8^ However, in a 2022 survey of hand surgeons, 12% reported using it.^11^ Overall, PPB excision, which includes traction neurectomy and suture closure, is a much more extensive process than skin tag removal. Therefore, the authors prefer to use code 26587 with a reduced service modifier (52), rather than codes 11420 or 11200, to adequately account for the technical skill and resources required for the procedure. In the future, a more appropriately weighted, specific CPT code for this procedure is required.

This study had certain limitations, including the retrospective nature of data collection and the lack of long-term outcomes and patient satisfaction data. Although surgical complications noted during follow-up visits were analyzed in this study, many patients who undergo office excision do not return to the clinic for scheduled follow-up. Patients could therefore have had unreported complications or dissatisfaction with the functional or cosmetic outcome of their procedure. Our study also did not consider patients’ insurance coverage, which may have added context to CPT code usage. Finally, detailed data on clinic facility charges were not available for analysis. If included, these charges would increase the total cost associated with clinic-based excisions. However, even with these additional costs, the total charges for clinic excision would likely remain minimal compared with the overall charges associated with operating room procedures.

Therefore, this study highlights the superior cost efficacy of office PPB excision. Although there was no considerable difference in outcomes between operating room and office excisions, operating room excisions had higher charges and reimbursements. Therefore, office excision should be performed when possible, with operating room excisions limited to patient or parent-specific factors, lack of clinic resources such as an adequate procedure room, or with concurrent procedures requiring general anesthesia. The results also showed variability in CPT code usage, and the authors believe that code 26587 with a reduced service modifier (52) would most accurately represent the skill and resources required for PPB excision.

## Conflicts of Interest

Dr Mendenhall is an educational consultant for PolyNovo, which does not relate to the content of this article. No benefits in any form have been received or will be received by the other authors related directly to this article.
